# Immobilised Histidine Tagged *β*_2_-Adrenoceptor Oriented by a Diazonium Salt Reaction and Its Application in Exploring Drug-Protein Interaction Using Ephedrine and Pseudoephedrine as Probes

**DOI:** 10.1371/journal.pone.0094955

**Published:** 2014-04-18

**Authors:** Qian Li, Liujiao Bian, Xinfeng Zhao, Xiaokang Gao, Jianbin Zheng, Zijian Li, Youyi Zhang, Ru Jiang, Xiaohui Zheng

**Affiliations:** 1 College of Life Sciences, Northwest University, Xi'an, Shaanxi, China; 2 Institute of Analytical Science, Northwest University, Xi'an, Shaanxi, China; 3 Institute of Vascular Medicine, Peking University; Third Hospital and Key Laboratory of Molecular Cardiovascular Sciences, Ministry of Education, Beijing, China; 4 School of Pharmacy, The Fourth Military Medical University, Xi'an, Shaanxi, China; Russian Academy of Sciences, Institute for Biological Instrumentation, Russian Federation

## Abstract

A new oriented method using a diazonium salt reaction was developed for linking *β*_2_-adrenoceptor (*β*_2_-AR) on the surface of macroporous silica gel. Stationary phase containing the immobilised receptor was used to investigate the interaction between *β*_2_-AR and ephedrine plus pseudoephedrine by zonal elution. The isotherms of the two drugs best fit the Langmuir model. Only one type of binding site was found for ephedrine and pseudoephedrine targeting *β*_2_-AR. At 37 °C, the association constants during the binding were (5.94±0.05)×10^3^/M for ephedrine and (3.80±0.02) ×10^3^/M for pseudoephedrine, with the binding sites of (8.92±0.06) ×10^−4^ M. Thermodynamic studies showed that the binding of the two compounds to *β*_2_-AR was a spontaneous reaction with exothermal processes. The ΔG^θ^, ΔH^θ^ and ΔS^θ^ for the interaction between ephedrine and *β*_2_-AR were −(22.33±0.04) kJ/mol, −(6.51±0.69) kJ/mol and 50.94±0.31 J/mol·K, respectively. For the binding of pseudoephedrine to the receptor, these values were −(21.17±0.02) kJ/mol, −(7.48±0.56) kJ/mol and 44.13±0.01 J/mol·K. Electrostatic interaction proved to be the driving force during the binding of the two drugs to *β*_2_-AR. The proposed immobilised method will have great potential for attaching protein to solid substrates and realizing the interactions between proteins and drugs.

## Introduction

The interactions between a protein and a drug are of growing interest due to the role in elucidating protein functions, explaining drug action mechanisms and discovering novel drug candidates [1], [2]. G-protein coupled receptors (GPCRs) are the largest and most diverse superfamily of integral membrane receptors due to their crucial role in curing varied diseases [3]–[5] and being the most important class of drug targets [6].

Currently, a wide range of methods have been developed for investigating the drug-protein interaction, including ultrafiltration [7], mass spectrometry [8], X-ray crystallography [9], nuclear magnetic resonance [10], surface plasmon resonance [11], affinity capillary electrophoresis [12] and computer-aided methods [13]. Although contributing greatly to the exploration of drug-protein interactions, these approaches need to be improved to overcome the limitations of long analysis time, requirements for samples with high concentrations or purities, potential interfering substance from the sample and relatively specialized equipment [14]. High performance affinity chromatography (HPAC) is another widely used method for the study of drug-protein interaction [15]. The method often involves immobilization of a protein on a solid matrix to construct affinity stationary phase.

Synthesising an assay for attaching a protein to a solid matrix is a key factor for accomplishing an HPAC method. Physical absorption and random immobilised methods are commonly reported ways to synthesis an affinity stationary phase [16], [17]. The types of stationary phases constructed by these methods have been confirmed to generate the inevitable issue of losing bioactive binding sites of the protein during the immobilisation procedure. Oriented immobilisation has proved feasible to address this issue in previous publications [18], [19] and should be further used to synthesise HPAC stationary phases attributed to the particularly important role in life sciences [20], [21].

**Figure 1:**
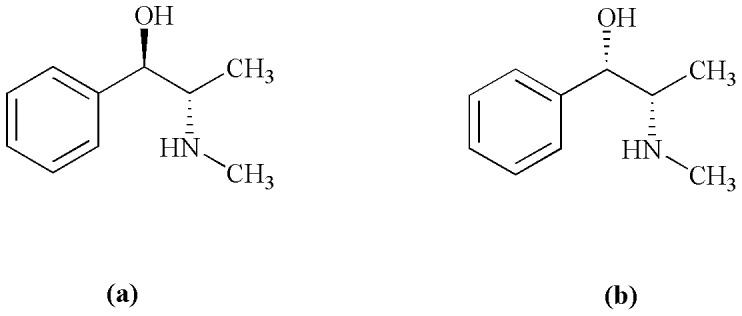
Chemical structures of ephedrine and pseudoephedrine. (a), *1R,2S*-ephedrine; (b), *1S,2S*-pseudoephedrine.

Beta_2_-adrenoceptor (*β*_2_-AR), one of the members of the GPCRs, is a crucial target of many drugs for fighting obesity and ailments of the heart and respiratory system [22]. Ephedrine and pseudoephedrine (Figure 1) are isomers widely used for treating diseases of the respiratory system. Both ephedrine and pseudoephedrine exert their therapeutic action through the same signal pathway involving *β*_2_-AR [23]. The two drugs have varied potency *in vivo* although they have similar structures and the same acting mechanism. One reason for this may be the different binding affinities of the two ligands to *β*_2_-AR. This work is firstly designed to establish an oriented immobilised method for fixing his-tagged fusion *β*_2_-AR on a solid matrix to synthesise an HPAC stationary phase. Further work is performed to investigate the binding behaviours of ephedrine and pseudoephedrine to *β*_2_-AR by HPAC methods.

## Theory

### Zonal elution

The binding of ephedrine and pseudoephedrine to the oriented immobilised *β*_2_-AR was determined by zonal elution. The theory assumes that a known concentration of a competing agent (I) continuously passes through a column containing an immobilised ligand (L) while the injecting volume of an analyte (A) is small enough to be neglected compared with the concentration of I in the mobile phase. Assuming I and A have direct competition at a single binding site on L and the interactions have association/dissociation kinetics, Eq. (1) can be used to calculate the binding of A and I to L [24], [25].
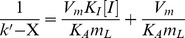
(1)where X is the capacity factor for A due to the existence of a second type of binding site on the column, and *k*' is the overall capacity factors of A ascribed to all the types of sites. In this case, the equation assumes the capacity factors of A produced by the individual sites are additive, and an independent estimate of the capacity factors due to the second type of site can be obtained, allowing for the capacity factor attributed to the first type of site 1 (Site 1) to be measured by *k*_1_' = *k*'-X.

When the column is equilibrated with an eluent at concentration [A] and [I] equal to zero, and the retention of compound A is injected as a perturbation, Eq. (2) is used to determine the binding of A to L:
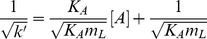
(2)


Assuming a single type of binding site, a linear expression can be obtained by plotting 1/sqrt (k') as a function of [A] to determine m_L_ and *K*_A_. For two types of binding sites, a second term is added to the k' expressions.

### Thermodynamic study

The thermodynamic interaction between *β*_2_-AR and ephedrine plus pseudoephedrine was described by the van't Hoff equation, listed as Eq. (3) and (4).
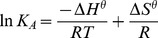
(3)

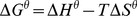
(4)


In these equations, R is the ideal gas constant (8.31 J/mol/K), and T is the temperature in Kelvin. According to the semi-empirical rules summarised by Ross et al. [26], when ΔG^θ^<0, ΔH^θ^>0 and ΔS^θ^>0, hydrophobic interaction is the main interaction force during the binding process of a drug to protein. If ΔG^θ^<0, ΔH^θ^<0 and ΔS^θ^>0, electrostatic interactions mainly contribute to the interaction. If ΔG^θ^<0, ΔH^θ^<0 and ΔS^θ^<0, Van der Waals forces or hydrogen bonds are the major forces of the binding.

## Materials and Methods

### Chemicals and instruments

Ephedrine (1*R*,2*S*-ephedrine, 171241) and pseudoephedrine (*1S*,*2S*-pseudoephedrine, 171237) were obtained from the National Institutes for Food and Drug Control (Beijing, China). Fatty acid and globulin free bovine serum albumin (BSA, A0281) was obtained as lyophilized powder from Sigma-Aldrich (St. Louis, MO, USA). The Quaternary Sepharose Fast Flow pre-packed column (16×25 mm), Ni^2+^ Sepharose 6 Fast Flow (10×58 mm) and Sephadex G25 pre-packed column (10×100 mm) were purchased from GE Healthcare Life Sciences (Uppsala, Sweden). Macroporous silica gel (SPS 300-7, pore size 300 Å, particle size 7.0 mm) was from Fuji Silysia Chemical Company Limited (Tokyo, Japan).

The HPLC system employed in this work consisted of a binary pump, a column oven from Agilent Technologies (Santa Clara, USA) and a diode array detector (Waldbronn, Germany). The data were collected and processed by a Chemstation 5.2 software installation.

### Expression and purification of *β_2_*-AR

The full gene of Sus scrofa *β*_2_-AR (Refseq:MN_ 001128436) was cloned in our previous work [27] and the protein was expressed in *E.coli* BL21 (DE3) with an N-terminal His-tag. The transformed *E. coli* strain was grown overnight at 37 °C in 50 mL Luria-Bertani (LB) medium containing 100 µg/L penicillin. When the value of OD_600_ reached 0.4–0.6, isopropyl-*β*-D-thiogalactopyranoside was added to a final concentration of 2.0 mM, and the culture was incubated at 30°C for 6.0 h. The cells containing His-tagged *β*_2_ *-*AR were separated from the culture media by centrifugation at 6000×g for 30 min at 4.0 °C, and the supernatant was discarded. Falcon tubes containing the cell pellets were placed into a dry ice/ethanol bath for 3–5 min and were transferred to cold water for 60 s to melt the ice along the walls of the tubes. The pellet was broken free by a sterile plastic spatula and suspended in 200 mL neutral lysis buffer (50 mM Tris-HCl, pH 7.4, containing 0.3 M NaCl and 0.5 mM EDTA) in a 500-mL flask. Lysozyme (0.5 mg/mL) was added to the buffer and incubated for 2.0 h on ice with agitation to digest the cell wall. The *E. Coli* cells were lysed using 20 mL of a 10× Mg/Mn salt solution (100 mM MgCl_2_ and 10 mM MnCl_2_). DNA was removed by DNase (10 µg/mL) with incubation for 30 min at RT. The resulting *E. coli* lysate (approximately 260 mL) was placed into a dialysis bag (10 kD molecular weight cut-off) in 4 L buffer (10 mM Tris-HCl, pH 7.4, containing 0.5 mM EDTA). The dialysis buffer was changed twice over a 16–24 h period. At the end of the dialysis, the cell debris was removed as a pellet by centrifugation at 10000× g for 30 min at 10 °C.

Dialysed and centrifuged recombinant *β*_2_-AR supernatant was applied to a Ni^2+^ Sepharose 6 Fast Flow column that had been equilibrated with dialysis buffer (10 mM Tris-HCl, pH 7.4, containing 0.5 mM EDTA). The column was eluted with an increasing salt gradient of up to 500 mM imidazole in buffer A (20 mM potassium phosphate, pH 7.4, containing 500 mM NaCl) at a speed of 1.0 mL/min. The fraction of interest was collected by eluting the column using 50% buffer B (buffer A in the presence of 500 mM imidazole, pH 7.4). Further purification of the collected fraction was performed on the Quaternary Sepharose Fast Flow column equilibrated with 18% buffer C (buffer A containing 800 mM NaCl, pH 7.4). The *β*_2_-AR solution was collected by gradient elution from the column using buffer C ranging from 18%–40% with a flow rate of 5.0 mL/min. The solution was finally desalted by the Sephadex G25 column.

### Oriented immobilisation of *β_2_*-AR

**Figure 2:**
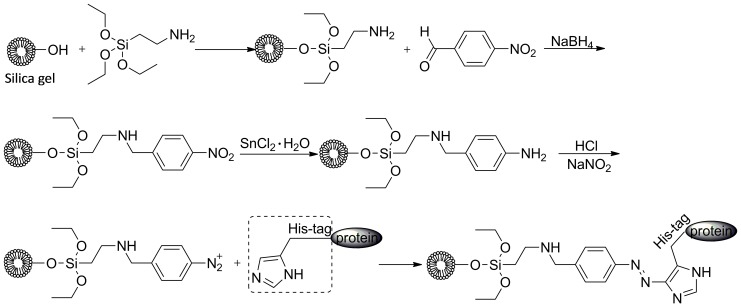
Diagram for oriented attaching His-tagged *β*_2_-AR on the surface of diazonium salt coated silica gel.

The *β*_2_-AR was oriented immobilised on macroporous silica gel using a diazo coupling method (Figure 2). In detail, aminopropyl-bonded silica was synthesised according to the method in our previous report [28]. Two grams of aminopropyl-bonded silica gel was suspended in a *p*-nitrobenzaldehyde methanol solution (18.2 mg/L, 100 mL) through stirring for 48 h at 60 °C. The resulting gel was filtrated by a sintered filter funnel and washed three times with methanol. The remnant methanol was removed by drying the gel in a vacuum oven at 60 °C for 12 h. The double bond in the produced gel was reduced to a single bond through reaction between the gel and 100 mL 8.6 µg/mL NaBH_4_ at room temperature for 12 h [29], using water as a solvent. After being filtrated and washed three times by water, the product was dried until the weight remained constant at 110 °C under vacuum. The nitro group in the gel was reduced to an amino group by mixing the gel with a SnCl_2_ ethanol solution (8.1 mg/L, 100 mL) and reacting for 6 h at 90 °C. The suspension was cooled to 0 °C and alkalified with 50 mL 5% NaHCO_3_, followed by a sequential cleaning with saturated NaCl and distilled water [30]. The produced phenylamine group in the gel was then transformed to its diazonium form through reaction with excess HCl (20 mM, 30 mL), in which a freshly prepared NaNO_2_ solution (20 mM) was added dropwise until reaching the turning point of potassium iodide starch paper changing to blue [31]. The whole procedure for preparing the diazonium salt was performed at 4 °C, and a post stirring for 10 min was needed to accomplish the reaction. The purified His-tagged *β*_2_-AR was immobilised on the resulting gel by immediately adding the receptor (61.67 µg/mL, 10 mL) solution into the suspension and reacting for 2 h at 4 °C. Finally, the gel was washed three times with phosphate buffer (20 mM, pH 7.4).

### Chromatography

The column containing the oriented immobilised *β*_2_-AR was prepared by packing the stationary phase into a stainless steel column (2.1 mm×5.0 cm) using the slurry method at a pressure of 4.0×10^7^ Pa. Phosphate buffer (20 mM, pH 7.4) was used as the slurry solution. The control column containing exposed silica gel and attached histidine was prepared using the same synthesis method as that of the *β*_2_-AR column.

The mobile phases were prepared by adding 0 to 40 µM of ephedrine hydrochloride as the competing agent to 20 mM phosphate buffer (pH 7.4). All of the mobile phases were filtrated with 0.45 µm Millipore HV filters and degassed by ultrasonication before HPAC analysis. Working solutions of ephedrine and pseudoephedrine were prepared by dissolving the drugs in phosphate buffer (20 mM, pH 7.4) to a concentration of 1.0 mM. The detection wavelength was 220 nm with an injection of 5.0 µL for each drug. The flow rate was 0.2 mL/min for all of the analyses. Under the proposed chromatographic conditions, the void time was 1.6 min using sodium nitrite as the injection solute. The capacity factor (*k*') was calculated by detecting the retention time in triplicate for each injected sample using the equation *k*' = (t_R_−t_0_)/t_0_. When the injection solute was pseudoephedrine, Eq. (1) was used to plot the curve of 1/(*k*'-X) and [A], whereas Eq. (2) was used to achieve the plot between 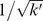 and [A] when ephedrine was used as the analyte.

### Thermodynamic study

The thermodynamic investigation of ephedrine and pseudoephedrine on oriented immobilised *β*_2_-AR was performed at 10, 20, 30, 37 and 45 °C. Eq. (3) and (4) were used to calculate the free energy, enthalpy and entropy during the binding of the two drugs to the receptor. The semi-empirical rules by Ross et al. [26] were utilised to reveal the binding mechanism process of the two drugs to *β*_2_-AR.

### Molecular docking

The sequence of Sus scrofa *β*_2_-AR (Accession Number: AF000134) was obtained from Genbank. The query sequence of the target receptor was searched find out the related protein structure to be used as a template by the BLAST (Basic Local Alignment Search Tool) program [32] against Protein Data Bank database. The three dimensional homology model was calculated using crystal structural coordinate of template on the basis alignment of target and template sequence of *β*_2_-AR according to the procedures illustrated by Discovery Studio 2.5 (DS 2.5, Accerlrys Software Inc., San Diego, CA). Structural evaluation of the receptor was performed using two programs named PROCHECK and Verify 3D. Once the three dimensional model was generated, energy minimisation was carried out for the receptor before dockings. Structure of ephedrine and pseudoephedrine were constructed by DS 2.5. CDOCKER program, implemented in the software platform of DS, was used for the docking study. The pose cluster radius was set to 0.5 with a value of top hits as 10.

## Results and Discussion

### Purification of his-tagged *β_2_*-AR

**Figure 3:**
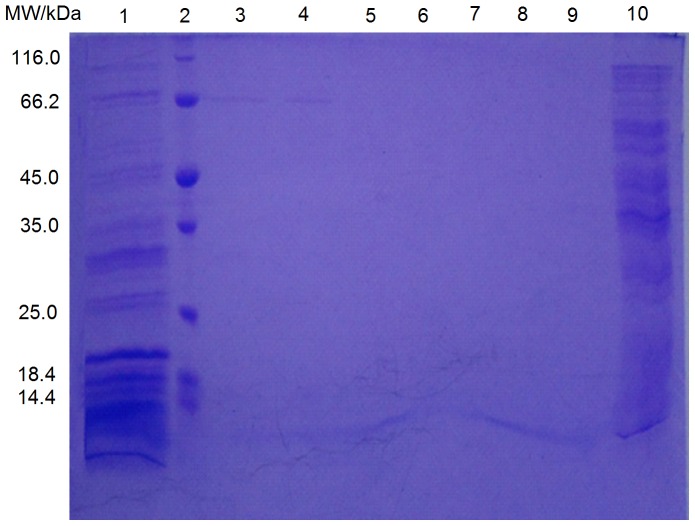
SDS-PAGE analysis of *β*_2_-AR solution. Lane 1, collection from Ni^2+^ Sepharose 6 Fast Flow column eluted by 50% buffer B; Lane 2, protein molecular weight marker (the molecular weights from top to bottom are 116.0, 66.2, 45.0, 35.0, 25.0, 18.4 and 14.4 kDa, respectively); Lane 3–4, collection from Quaternary Sepharose Fast Flow column gradient eluted by 18%–40% buffer C; Line 5–9, collection from Quaternary Sepharose Fast Flow column eluted by 50%, 60%, 70%, 80% and 90% buffer C; Lane 10, *β*_2_-AR supernatant.

The total protein in the desalted *β*_2_-AR solution was determined by bicinchoninic acid (BCA). Using BSA as a standard, the calibration curve was plotted to assay the total protein in the solution. The linear regression equation was y = 0.0002x+0.2702, with a correlation coefficient of 0.9957. The concentration of the total protein in the *β*_2_-AR solution was (61.67±4.48) µg/mL. Sodium dodecyl sulphate polyacrylamide gel electrophoresis (SDS-PAGE) was used to detect the molecular weight of the protein in the desalted *β*_2_-AR solution (Figure 3). The lanes of the desalted *β*_2_-AR solution presented only one clear band corresponding to the band of the marker with a molecular weight of 66.5 kDa, based on which the molecular weight of the protein in the desalted *β*_2_-AR solution is believed to be 66.5 kDa [30]. The purity of *β*_2_-AR in the solution was calculated to be 95% of the total protein by densitometry quantification of the lane using the BandScan software. Furthermore, the desalted *β*_2_-AR solution was also analysed by exclusion-size chromatography, which did not present any interference of the peak with a molecular weight of 66.5 kDa and gave a purity of 96% for the peak corresponding to *β*_2_-AR. Taken together, the concentration of *β*_2_-AR was approximately 59 µg/mL.

### Immobilisation of His-tagged *β_2_*-AR

Random and oriented immobilised methods are the main assays for attaching a protein to solid substrates. Comparing with random methods, the proposed approach is oriented, and thus, it had the properties of better maintaining the bioactive characteristics and increasing the binding sites of the protein [33]. The concentration of the immobilised His-tagged *β*_2_-AR on silica gel was calculated by detecting the free concentration of *β*_2_-AR in the reaction solution before and after the diazonium reaction through the bicinchoninic acid method. The amount of *β*_2_-AR attached on the gel was 200.17±7.47 pmol/g. This value was much greater than for the random immobilisation reported earlier [24], [33]. The commonly reported oriented method for protein immobilisation included the use of metal chelate resin [34] and horseradish peroxidase [35]. This work developed a new oriented method through the reaction between the histidine tag in the protein and the diazonium salt activated silica gel. Compared with the previous two types of oriented methods, the proposed assay had more potency in varied protein immobilisation, attributed to the lack of structure selectivity of the protein.

### Specificity and stability of *β_2_*-AR column

**Figure 4:**
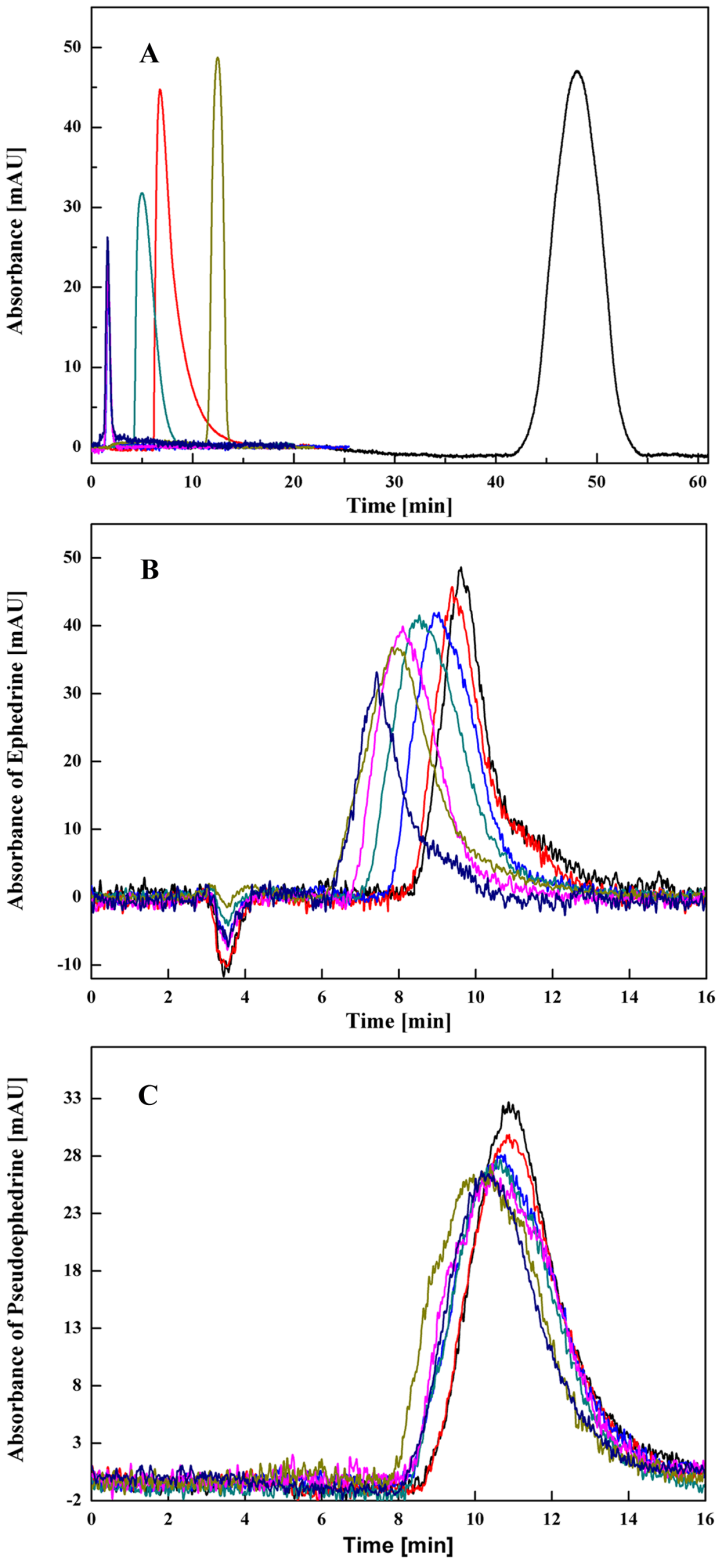
Representative chromatograms of control drugs, ephedrine and pseudoephedrine. A, Control drugs were eluted from *β*_2_-AR column using 20 mM phosphate buffer (pH 7.4) as mobile phase at 0.2 mL/min. Non-retained peaks: sodium nitrite, terazosin, esmolol. Retained peaks: salbutamol, clorprenaline, tobuterol, bambuterol (left to right); B, Ephedrine eluted from *β*_2_-AR column when the mobile phase containing ephedrine at different concentration (from right to left: 4, 8, 12, 20, 24, 30 and 34 µM); C, Pseudoephedrine eluted from *β*_2_-AR column when the mobile phase containing ephedrine at different concentration (from right to left: 2, 4, 8, 12, 16, 24 and 30 µM).

The specificity of the oriented *β*_2_-AR column was tested by determining the retention times of specific drugs on the receptor. The retention times of the specific ligands, including salbutamol, clorprenaline, tobuterol and bambuterol were 5.0, 9.9, 12.5 and 49.1 min, respectively, whereas the specific ligands of the *β*_1_-adrenoceptor and *α*_1_-adrenoceptor, esmolol and terazosin, both gave retention times of 1.6 min on the column (Figure 4A). The varied retention times illustrated that His-tagged *β*_2_-AR still had the specificity for recognising its ligands after the immobilisation and packing procedures. Meanwhile, the rank order of the retention times of salbutamol, tulobuterol and clenbuterol is in line with the order of their association constants calculated by the radio-ligand binding assay [36]. These data confirms that it is reasonable to characterise the *β*_2_-AR column using the retention times of the ligand targets of the receptor.

The stability of *β*_2_-AR was investigated by detecting the retention times of salbutamol, clorprenaline and tobuterol for 30 consecutive days. The relative standard deviations (RSD) of the retention times on those days were 2.43%, 1.48% and 3.13%, indicating that the retention times of the drugs remained stable over the 30 days. The peak profiles were also stable over the 30 days. In conclusion, the *β*_2_-AR column had good stability for at least 30 days. The specific drugs of *β*_2_-AR give retention times approximate to the void time (Table 1) on the control column, which indicates that the retention of the specific ligands on the *β*_2_-AR column is a consequence of the *β*_2_-AR presentation but not a nonspecific interaction with the silica gel.

**Table 1:** Retention times of *β*_2_-AR specific ligands on different columns.

Ligand	Retention times (min)		
	Exposed silica gel	Histidine coated gel	Immobilized *β*_2_-AR
Salbutamol	1.65	1.62	5.0
Tobuterol	1.58	1.67	9.9
Clorprenaline	1.71	1.64	12.5
Bambuterol	1.56	1.59	49.1

Notes: All the capacity factors are determined triplicate at 37 °C. The void time is measured as 1.6 min using sodium nitrite as an injection.

### Self competitive studies

**Figure 5:**
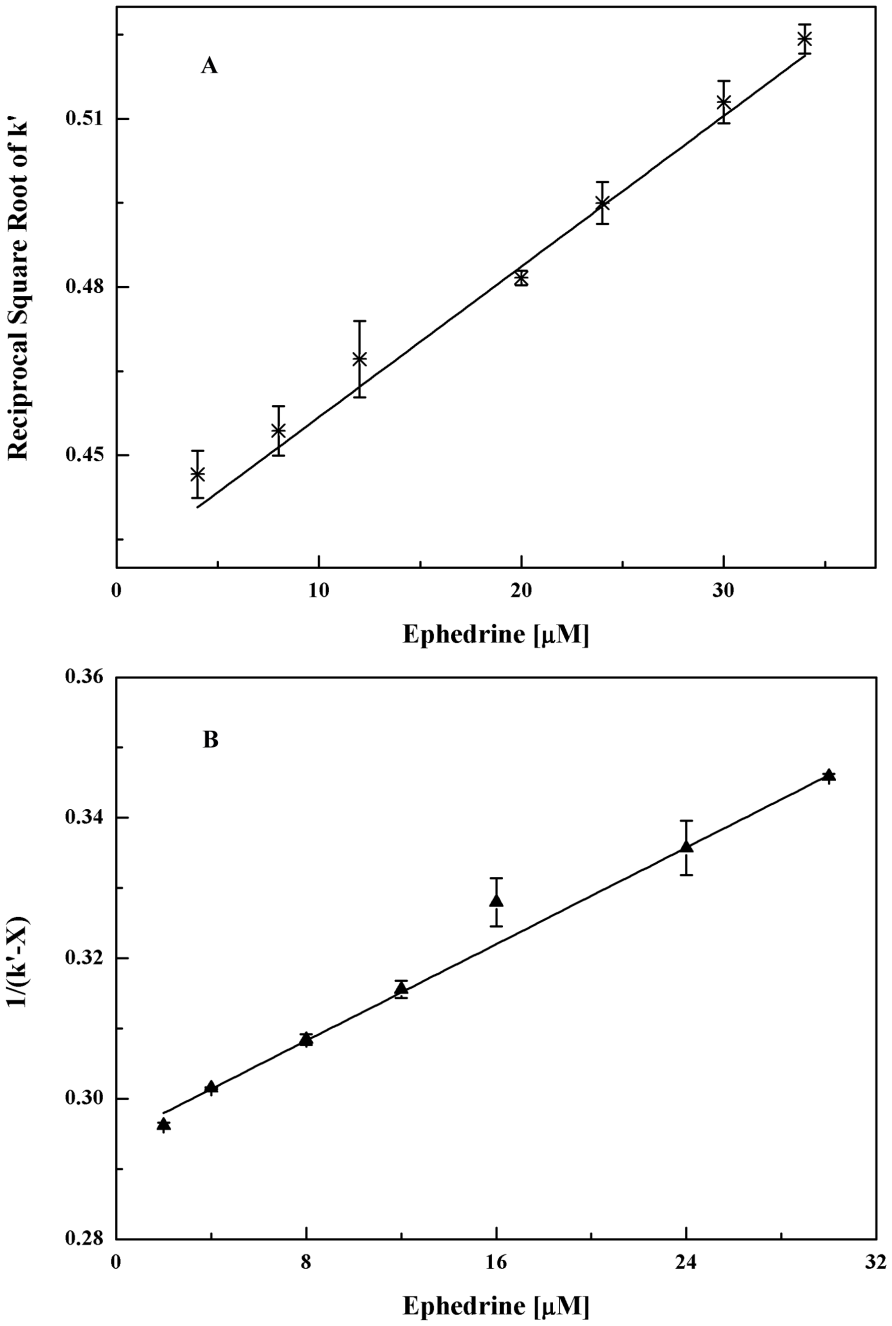
Change in 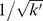 of ephedrine and 1/(k′-X) of pseudoephedrine with mobile-phase concentrations of ephedrine. Data were from an oriented immobilised *β*_2_-AR column at 37 °C. The equations of the best fit lines were y = (2.58±0.02)×10^3^x+(0.43±0.01) for ephedrine with a coefficient of 0.9962 and y = (1.76±0.07)×10^3^x+(0.29±0.01) for pseudoephedrine with a coefficient of 0.9915. The value of X used for the plot of pseudoephedrine was set at (2.49±0.26). A, Ephedrine at different concentrations was added in the mobile phase. The injection solute was 5.0 nmol of ephedrine; B, Ephedrine at different concentrations was added in the mobile phase while 5.0 nmol of pseudoephedrine was injected as analytes.

Self-competitive studies were performed where ephedrine served as both analyte and competing agent at 37 °C (Figure 4B). In this case, the injection volume of the analyte was 5.0 µL of 40 µM ephedrine, and the competitive agent in the mobile phase was 4, 8, 12, 20, 24, 30 or 34 µM ephedrine. Triplicate injections were performed for each concentrations of the competitive agent in the mobile phase. The capacity factors of ephedrine in the *β*_2_-AR column are summarised in Table 2. The capacity factors of ephedrine gave a negative response to the concentrations of ephedrine in the mobile phase. Meanwhile, the capacity factors did not reach saturated adsorption during the tested concentrations, providing a situation of the response in the linear scope of the isotherm curve. According to eq. (2), plotting the curve of 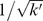 against the concentrations of ephedrine in the mobile phase at 37 °C (Figure 5A), a good linear relationship is obtained with a regression equation of y = (2.58±0.02)×10^3^x+(0.43±0.01) and a correlation coefficient of 0.9962 over the seven data points, providing a proof of one type of binding site for ephedrine binding to *β*_2_-AR. Based on which ephedrine is believed to have only one type of binding site on the *β*_2_-AR column. By dividing the intercept by the slope of Figure 5A, the association constant of ephedrine on the column was calculated to be (5.94±0.05)×10^3^/M (Table 3). Through the slope of Figure 5A and the values of the association constant, the concentration of the binding site for ephedrine targeting *β*_2_-AR was estimated to be (8.92±0.06) ×10^−4^ M.

**Table 2:** The capacity factors of ephedrine and pseudoephedrine at 37 °C.

Ephedrine (µM)	*k*′e	Ephedrine (µM)	*k*′p
4	5.01±0.02	2	5.87±0.01
8	4.84±0.02	4	5.81±0.01
12	4.58±0.03	8	5.73±0.01
20	4.31±0.01	12	5.66±0.01
24	4.08±0.01	16	5.54±0.03
30	3.80±0.01	24	5.47±0.03
34	3.64±0.01	30	5.38±0.01

Notes: *k*′e and *k*′p represent the capacity factors of ephedrine and pseudoephedrine respectively. *k*′e is determined when ephedrine serves both injecting solute and competitive agents with the concentrations of 4, 8, 12, 20, 24, 30 and 34 µM. While *k*′p is obtained by direct competitive study for which pseudoephedrine is used as an injection and ephedrine acts as competitive agents with the concentrations of 2, 4, 8, 12, 16, 24 and 30 µM.

**Table 3:** The association constants of ephedrine and pseudoephedrine binding to *β*_2_-AR at 37°C.

Injection analyte	
Ephedrine	Pseudoephedrine
K_A ephedrine_ = (5.94±0.05)×10^3^ M^−1^	K_A ephedrine_ = (5.98±0.24)×10^3^ M^−1^
	K_A pseudoephedrine_ = (3.80±0.02) ×10^3^ M^−1^

### Direct competitive study

A direct competitive study was conducted using ephedrine as a mobile phase additive and pseudoephedrine as an injection solute at 37 °C (Figure 4C). As shown in Table 2, a decreasing capacity factor of pseudoephedrine was found when the concentration of ephedrine in the mobile phase was increased. The capacity factors of pseudoephedrine were not saturated during the tested concentrations, which indicated that the pseudoephedrine absorption properties were in line with the Langmuir model. Eq. (1) was used to plot the curve between the pseudoephedrine capacity factors and the concentrations of ephedrine in the mobile phase. Using Eq. (1), a linear relationship was obtained between the 1/(*k*'*-X*) of pseudoephedrine and the concentrations of ephedrine, as shown in Figure 5B. The value of X was calculated to be 2.49±0.26 by non-linear regression. By applying the slope and intercept of the linear curve in Figure 5B, the association constant of ephedrine was determined to be (5.98±0.24)×10^3^/M, as shown in Table 3. This value was in good agreement with the association constant obtained by the self- competitive studies. According to the hypothesis of zonal elution assay, the concentration of the binding sites for pseudoephedrine was (8.92±0.06) ×10^−4^ M based on the value of ephedrine calculated by self-competitive studies. Using the binding sites and the intercept of Figure 5B, the association constant for pseudoephedrine binding to *β*_2_-AR was determined to be (3.80±0.02) ×10^3^/M, as shown in Table 3.

The association constant of ephedrine is greater than that of pseudoephedrine even though the structures are similar. The difference between the structures of the two drugs is the configuration of the amino group, which produces the different spatial selectivity of the two drugs to the binding sites on *β*_2_-AR during the interactions with the receptor. Although the association constants of the two drugs have the same order of magnitude, the exact values are varied due the high repeatability and precision of the HPAC assay, on which the spatial position of the amino groups are believed to contribute greatly to the binding of the two drugs and *β*_2_-AR. This explanation can be confirmed by the research of Vansal et al. [37] who found that *1S*,*2R*-ephedrine has a maximal response relative to isoproterenol (EC50) of 72 µM, whereas *1S*,*2S*-pseudoephedrine gives an EC50 value of 309 µM on *β*_2_-AR. Taken together, it is concluded that the varied affinity of the two drugs to *β*_2_-AR may be one of the key factors for their different pharmacological potency through the signal pathway involving *β*_2_-AR.

### Thermodynamic studies

**Figure 6:**
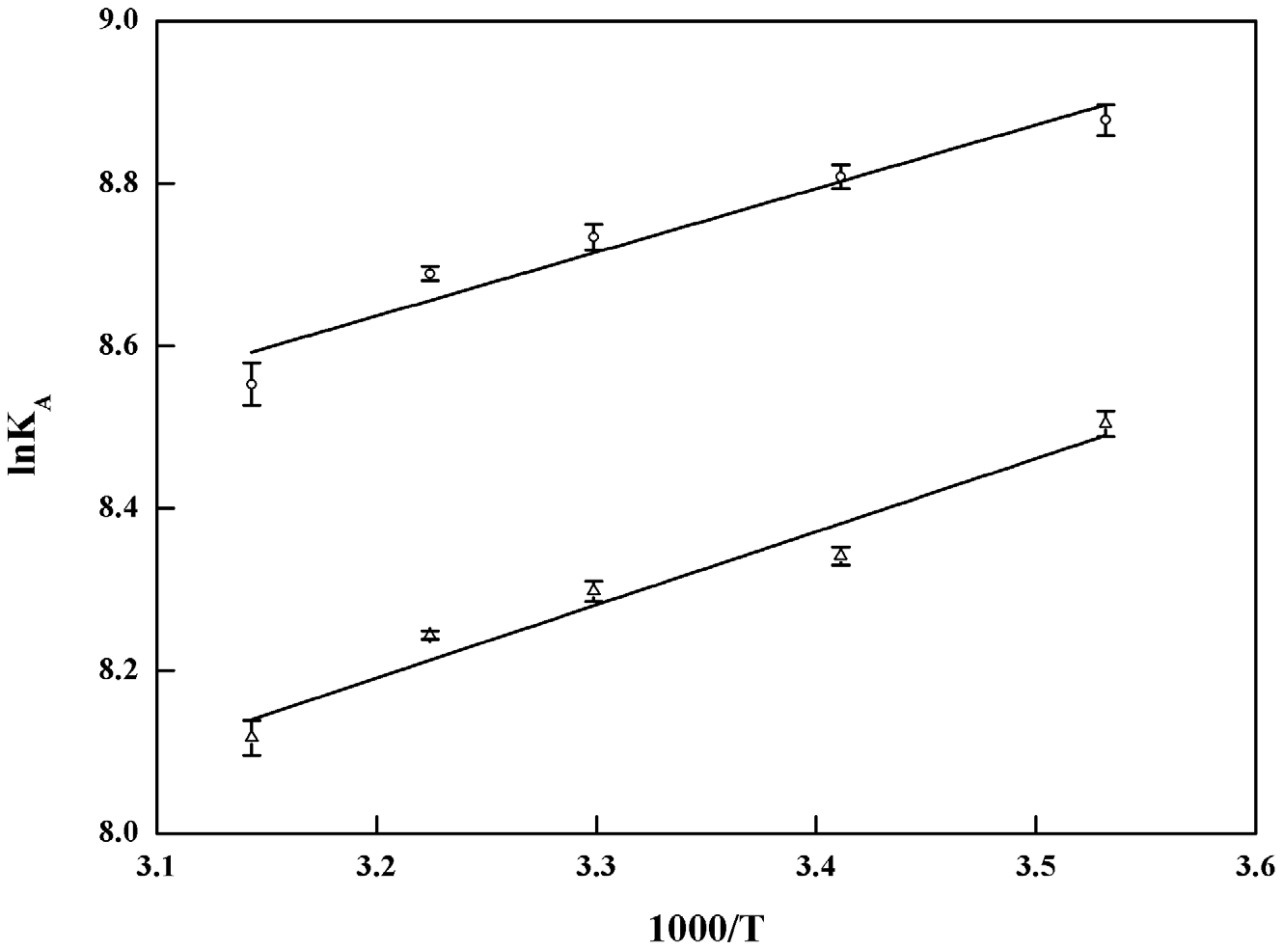
Van't Hoff plots for the interaction of ephedrine and pseudoephedrine binding to *β*_2_-AR. Circle, best-fit line with an equation of y = (0.78±0.08)x+(6.12±0.28) for ephedrine and a coefficient of 0.9716. Triangle, Best-fit curve for pseudoephedrine with an equation of y = (0.90±0.07)x+(5.31±0.23) and a coefficient of 0.9771. All the experiments were performed at 10, 20, 30, 37 and 45 °C.

Thermodynamic studies between the two drugs and *β*_2_-AR were performed at 10, 20, 30, 37 and 45 °C. Zonal elution was used to calculate the association constants of ephedrine and pseudoephedrine at those temperatures. According to eq. (3), linear relationships were found by plotting the lnK_A_ of ephedrine and pseudoephedrine against 1000/T, with correlation coefficients of 0.9716 and 0.9771 (Figure 6), respectively. The ΔH^θ^ and ΔS^θ^ for ephedrine binding to *β*_2_-AR were calculated to be −(6.51±0.69) kJ/mol and 50.94±0.31 J/mol·K. By eq. (4), the free energy during the binding was −(22.33±0.04) kJ/mol (Table 4), indicating a spontaneous reaction during the binding procedure. For the interaction between ephedrine and *β*_2_-AR, ΔH^θ^<0, ΔS^θ^>0 and ΔG^θ^<0, which confirmed an exothermal process synergistically controlled by increasing entropy and decreasing enthalpy. During the process, ΔH^θ^ contributed 29.15% to ΔG^θ^, whereas ΔS^θ^ accounted for 70.85% of the contribution to ΔG^θ^ at 37 °C. Electrostatic interaction was believed to be the main force of the binding of ephedrine to *β*_2_-AR.

**Table 4:** The free energy and association constants of ephedrine and pseudoephedrine in different temperature.

Temperature (K)	Ephedrine		Pseudoephedrine	
	Association constant (×10^3^ M^−1^)	ΔG^θ^ (kJ/mol)	Association constant (×10^3^ M^−1^)	ΔG^θ^ (kJ/mol)
283.15	7.17±0.14		4.94±0.08	
293.15	6.69±0.10	−(22.33±0.04)	4.19±0.05	−(21.17±0.02)
303.15	6.21±0.10		4.02±0.05	
310.15	5.94±0.05		3.80±0.02	
318.15	5.18±0.13		3.35±0.07	

The values of ΔH^θ^, ΔS^θ^ and ΔG^θ^ for the binding of pseudoephedrine to *β*_2_-AR were −(7.48±0.56) kJ/mol, 44.13±0.01 J/mol·K and −(21.17±0.02) kJ/mol (Table 4). Similar to the interaction between ephedrine and *β*_2_-AR, the binding of pseudoephedrine to the receptor was found to be a spontaneous reaction due to the negative value of ΔG^θ^. The binding interaction proved to be mainly driven by electrostatic forces because ΔH^θ^<0, ΔS^θ^>0 and ΔG^θ^<0 during the binding. The free energy for the binding of ephedrine and *β*_2_-AR is more negative than that of pseudoephedrine, indicating that ephedrine more easily targets *β*_2_-AR. This may be another reason for the fact that the two drugs have varied pharmacological potency.

The association constants of ephedrine and pseudoephedrine binding to *β*_2_-AR decreased with increasing temperature, whereas the binding sites presented a positive response to an increasing temperature. When the temperature changed from 10 °C to 45 °C, the association constant of the two drugs decreased from 7.17×10^3^/M to 5.18×10^3^/M and from 4.93×10^3^/M to 3.36×10^3^/M, respectively. Meanwhile, the binding sites increased from 8.07×10^−4^ M to 9.48×10^−4^ M during the interaction, and the capacity factors of the two drugs on the *β*_2_-AR column diminished from 5.40 to 4.63 and from 6.36 to 5.60 when the concentrations were 4.0 µM. The association constants decreased 1.4 and 1.5 fold, whereas the binding sites increased 1.2 fold. At the same time, the capacity factor diminished 1.2 fold. The retention behaviour of a solute on an affinity column ascribed to the synergistic contribution of the association constant and the binding sites. For ephedrine and pseudoephedrine binding to *β*_2_-AR, the decreasing retention is mainly attributed to the diminishing association constants. The reason for this phenomenon may be related to the conformation change induced by the temperature.

In the literature, it is reported that *β*_2_-AR has three types of binding sites. One is the salt bridge between the amine group of the ligand and the carboxylate side chain of Asp^113^ in the third hydrophobic domain. The other two types of binding interactions proceed through hydrogen bonds between the hydroxyl side chain of Ser ^204^ and the *meta*-hydroxyl group of the ligand or between the hydroxyl side chain of Ser ^207^ and the *para*-hydroxyl group of the ligand [38]. Ammonium salts mainly interact with the first type of binding site [39]. Being members of the group of ammonium salts, electrostatic interaction is believed to be the driving force during the binding of the two drugs to *β*_2_-AR. In this presentation, both ephedrine and pseudoephedrine gave a thermodynamic profile of ΔG^θ^<0, ΔH^θ^<0 and ΔS^θ^>0. This result confirmed that electrostatic interaction is the main force during the bindings. Thermodynamic studies in this work illustrated the same point to aforementioned report, which paves the way to the application of the thermodynamic method in revealing the binding mechanism of the interaction between ligands and the receptor.

As noted in the literature [37], stereoselective and rank differences exist among the direct effects of ephedrine isomers on *β*_2_-AR; 1*R*,2*S*-ephedrine is the most potent. In this case, the affinity of 1*R*,2*S*-ephedrine binding to *β*_2_-AR should be stronger than the other isomers. In this work, the association constants of 1*R*,2*S*-ephedrine and 1*S*,2*S*-pseudoephedrine were calculated as (5.94±0.05)×10^3^ M^−1^ and (3.80±0.02) ×10^3^ M^−1^, respectively. This result is notable ascribed to the high agreement with aforementioned report. Waldeck et al [39] has discovered that ephedrine has a marked positive inotropic effect on the papillary muscle with a pD_2_ of about 5.95, which corresponds an dissociation constant of 11200 nM for the drug binding to *β*_2_-AR. Subsequent studies by Samama et al [40] and Seifert et al [41] have reported an dissociation constant of 8000 nM for 1*R*,2*S*-ephedrine binding to wild-type *β*_2_-AR and a value of 5100±1300 nM to *β_2_*-ARGsα_S_. Based on the association constant in this work, the dissociation constant for 1*R*,2*S*-ephedrine to the receptor was 168000 nM. Since the receptors used in these work are cloned with various gene sequence, it is expected that the exact values of dissociation constant for the drug differ from each other.

### Molecular docking

**Figure 7:**
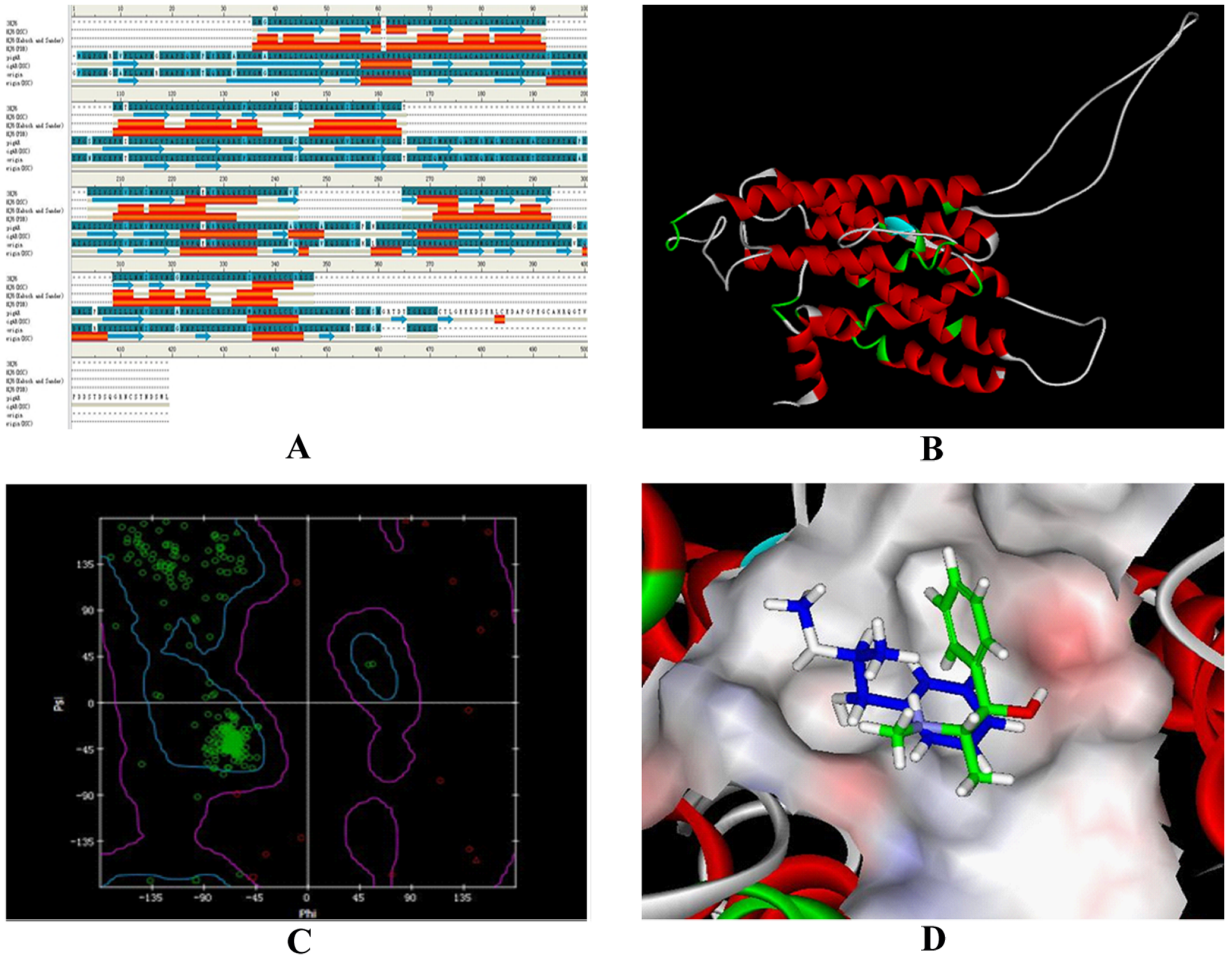
Three dimensional model of *Sus scrofa β*_2_-AR by homology modelling and its binding to ephedrine and pseudoephedrine. A, Structural-based alignment of *β*_2_-AR (PDB ID: 3KJ6). The sequence shaded in cyan represents sequence similarity. Red colour means the transmembrane domain; B, The best predicted model of *β*_2_-AR (pigAR.BL00010001); C, Ramachandran plot of the *β*_2_-AR model calculated by PROCHECK; D, Docking complexes overview of ephedrine/*β*_2_-AR and pseudoephedrine/*β*_2_-AR. Green illustrates Ephedrine. Blue illustrates Pseudoephedrine.

One template had been identified (PDB ID: 3KJ6, methylated *β*_2_-AR-Fab complex) through homology searches in PDB by comparing and aligning the target sequence with that in PDB. The sequence identity was 50%. Followed by structural-based alignment between query and target receptor, C-alpha atoms were selected for the model building ascribed to the highest identity (Figure 7A). Under the desired modelling condition, ten annotated model structure of the receptor were predicted. The best model structure of *β*_2_-AR superimpose to the template was believed to be pigAR.BL00010001 (Figure 7B). It showed the lowest value in PDF (Probability Density Functions) Total Energy (−2866.93), PDF Physical Energy (−5004.22) and DOPE (Discrete Optimized Protein Energy) score (−31734.13). These results indicated that the best model of *β*_2_-AR compared to the other models. The structural evaluation of *β*_2_-AR model revealed that sterochemical and geometrical parameters incorporated in PROCHECK were satisfied in this model. The Ramachandran Plot of the receptor model calculated by PROCHECK was showed in Figure 7C. It was notable that most of the amino acid residues of *β*_2_-AR distributed in the favoured region. Further verification of the model by Verify 3D confirmed that a good consistent of *β*_2_-AR with the respective amino acid due to 65.30% of the residues having an averaged compatibility of an atomic model (3D) with its own amino acid sequence (1D).

Site directed docking results showed that only one type of binding domain is engaged in the interaction between the two drugs and *β*_2_-AR (Figure 7D). Asp ^79^ (corresponding to the reported Asp ^113^ of the reported *β*_2_-AR) proved to be the exact site for the two isomers binding to the receptor. Meanwhile, no hydrogen bond was found during this interaction. It is widely known that Asp is an acidic amino acid with an isoelectric point of 2.98. In this case, Asp possesses negative charge in a solution with pH of 7.4. While in the same environment, both ephedrine and pseudoephedrine exist as positive ions. Based on these points, we concluded that the binding of the two drugs to *β*_2_-AR is driven by electrostatic interaction. This result is in good agreement with the results from thermodynamic investigation in this work and the report by Strader et al [42]–[43].

## Conclusion

A diazo coupling method was developed for oriented immobilising *β*_2_-AR onto the surface of macroporous silica gel. Compared with random immobilised assays, the current method proves to have the advantages of better maintaining the bioactive characteristics of *β*_2_-AR due to the greater amount of the immobilised protein on the gel and the increased binding sites on the surface of the receptor for recognising its ligands. The stationary phase prepared by the oriented method was also applied in exploring the interaction between *β*_2_-AR and two drugs, ephedrine and pseudoephedrine. The varied association constants of the two drugs are believed to be the key reason for their different pharmacological potency. The binding of the two drugs to *β*_2_-AR was mainly driven by the electrostatic interaction between the amino group in the structure of the drugs and the carboxylate side chain of Asp^113^ in the third hydrophobic domain of the receptor. The oriented method is potential in preparing stationary phases containing other receptors for HPAC to study drug-receptor interaction.
